# Reciprocal change in Glucose metabolism of Cancer and Immune Cells mediated by different Glucose Transporters predicts Immunotherapy response

**DOI:** 10.7150/thno.48954

**Published:** 2020-07-25

**Authors:** Kwon Joong Na, Hongyoon Choi, Ho Rim Oh, Yoon Ho Kim, Sae Bom Lee, Yoo Jin Jung, Jaemoon Koh, Samina Park, Hyun Joo Lee, Yoon Kyung Jeon, Doo Hyun Chung, Jin Chul Paeng, In Kyu Park, Chang Hyun Kang, Gi Jeong Cheon, Keon Wook Kang, Dong Soo Lee, Young Tae Kim

**Affiliations:** 1 Department of Thoracic and Cardiovascular Surgery, Seoul National University Hospital, Seoul, Republic of Korea.; 2 Department of Nuclear Medicine, Seoul National University Hospital, Seoul, Republic of Korea.; 3 Seoul National University Cancer Research Institute, Seoul National University College of Medicine, Seoul, Republic of Korea.; 4 Biomedical Sciences, Seoul National University College of Medicine, Seoul, Republic of Korea.; 5 Department of Pathology, Seoul National University Hospital, Seoul, Republic of Korea.

**Keywords:** tumor microenvironment, tumor metabolism, glucose transporter, immunotherapy, lung cancer, lung squamous cell carcinoma

## Abstract

The metabolic properties of tumor microenvironment (TME) are dynamically dysregulated to achieve immune escape and promote cancer cell survival. However, *in vivo* properties of glucose metabolism in cancer and immune cells are poorly understood and their clinical application to development of a biomarker reflecting immune functionality is still lacking.

**Methods:** We analyzed RNA-seq and fluorodeoxyglucose (FDG) positron emission tomography profiles of 63 lung squamous cell carcinoma (LUSC) specimens to correlate FDG uptake, expression of glucose transporters (GLUT) by RNA-seq and immune cell enrichment score (ImmuneScore). Single cell RNA-seq analysis in five lung cancer specimens was performed. We tested the GLUT3/GLUT1 ratio, the GLUT-ratio, as a surrogate representing immune metabolic functionality by investigating the association with immunotherapy response in two melanoma cohorts.

**Results:** ImmuneScore showed a negative correlation with GLUT1 (*r* = -0.70, *p* < 0.01) and a positive correlation with GLUT3 (*r* = 0.39, *p* < 0.01) in LUSC. Single-cell RNA-seq showed GLUT1 and GLUT3 were mostly expressed in cancer and immune cells, respectively. In immune-poor LUSC, FDG uptake was positively correlated with GLUT1 (*r* = 0.27, *p* = 0.04) and negatively correlated with ImmuneScore (*r* = -0.28, *p* = 0.04). In immune-rich LUSC, FDG uptake was positively correlated with both GLUT3 (*r* = 0.78, *p* = 0.01) and ImmuneScore (*r* = 0.58, *p* = 0.10). The GLUT-ratio was higher in anti-PD1 responders than nonresponders (*p* = 0.08 for baseline; *p* = 0.02 for on-treatment) and associated with a progression-free survival in melanoma patients who treated with anti-CTLA4 (*p* = 0.04).

**Conclusions:** Competitive uptake of glucose by cancer and immune cells in TME could be mediated by differential GLUT expression in these cells.

## Introduction

Cancer cells constitute cancer-permissive settings in the tumor microenvironment (TME) by inducing failure of the anti-tumor immunity 1. One of the mechanisms of cancer immune escape is the competitive metabolic interaction between cancer cells and immune cells in nutrient deprivation environments 2, 3. In particular, glucose, a key nutrient of the competition, is rapidly metabolized by enhanced aerobic glycolysis in cancer through the so-called Warburg effect 4. As immune cell switch from a quiescent to an activated status is also associated with enhanced glycolysis activation, the competitive interaction between cancer and immune cells could significantly reduce glucose availability to immune cells, thus hampering their antitumor immune functions 5, 6.

The status of immune cells in TME is a predictive biomarker for the antitumor efficacy of immune checkpoint inhibitors (ICI). The density of tumor-infiltrating lymphocytes is associated with good response to ICI and dynamically increased according to the ICI followed by tumor regression 7, 8. As activated immune cells including tumor-infiltrating CD8+ T-lymphocytes inhibit metabolic activity of cancer cells 9, the relative metabolic activity in cancer and immune cells can reflect the anti-tumor immune functionality. In this regard, evaluating the respective metabolic activity of immune cells and cancer cells may be a feasible biomarker to assess immune cell functionality in TME 10. In spite of the fact that the metabolic interaction in TME is a crucial factor involved in the anti-tumor immune functionality, such a biomarker that takes into account the metabolism of cancer and immune cells has not yet been discovered.

The high glucose consumption of malignant tumors requires increased glucose uptake mediated by facilitative glucose transporters (GLUT). The overexpression of GLUT, particularly GLUT1 and/or GLUT3 in tumors is commonly found across various cancer types 11-13. The overexpression of GLUT has been clinically applied and is now widely used for tumor imaging via Fluorodeoxyglucose (FDG) positron emission tomography (PET) 14, 15. While previous conventional studies estimate the GLUT1 and GLUT3 of tumors regardless of the heterogeneous cellular subpopulation of tumor tissue, here we scrutinize the expression of GLUT1 and GLUT3 in TME using single-cell RNA-sequencing (scRNA-seq) as well as tumor tissue RNA-sequencing (RNA-seq) data from lung cancer patients. FDG PET paired with the tumor RNA-seq of lung cancer patients allows evaluating *in vivo* functionality of glucose uptake associated with the immune status of TME as well as different GLUTs. By assessing the differential GLUT expression in cancer and immune cells, we investigated whether GLUT expression levels can be used as a biomarker to uncover immune functionality in TME using melanoma cohorts who underwent immunotherapy.

## Methods

### RNA-seq data paired with FDG PET of the study population

We evaluated RNA-seq profiles of 101 lung squamous cell carcinoma (LUSC) patients who underwent surgical resection between 2011 and 2013 16. Among them, 63 patients with preoperative FDG PET imaging which was taken in our institution and with tumor volume larger than 5.0 cm^3^ were included for the analysis of the association between FDG uptake and gene expression values. The details of RNA-seq of LUSC patients from our institution were described in previous study 16. The demographic and clinical characteristics of the study population are summarized in Table S1. The acquisition method of FDG PET data and the processing method of RNA-seq of LUSC patients to estimate glycolysis enrichment score are described in Supplementary Methods. The study protocol was reviewed by the institutional review board and approved as a minimal-risk retrospective study (approval no. H-1312-117-545) that did not require individual consent according to the institutional guidelines for consent waiver.

We also used mRNA transcriptome data of LUSC from The Cancer Genome Atlas projects (TCGA) and concordant FDG PET image from The Cancer Imaging Archive (TCIA) for validation purpose 17. Processing of TCGA and TCIA data for pairs of RNA-seq and FDG PET are described in Supplementary Methods.

### Estimating immune enrichment score

To evaluate the heterogeneous cellular landscape of TME, cell type enrichment scores were evaluated. A gene-signature based method for inferring 64 cell types from tissue transcriptome profiles, the xCell tool (http://xcell.ucsf.edu/), was used 18. xCell tool also reports an immune cell enrichment score, ImmuneScore, for each sample by summing estimated cell type enrichment scores of B-cells, CD4+ T-cells, CD8+ T-cells, dendritic cells, eosinophils, macrophages, monocytes, mast cells, neutrophils, and NK cells. Each immune cell enrichment score as well as ImmuneScore, the summed score of immune cells of the aforementioned cells, were estimated from transcripts of each study cohort.

The histogram of ImmuneScore was drawn, and it showed two peaks. Thus, LUSC were divided into two clusters, immune-poor, and immune-rich clusters. To determine the threshold to define clusters, kernel density estimation was applied to the histogram of ImmuneScore using R function '*density*'. The point of local minima was used as the threshold for the clusters.

### FDG PET analysis

To obtain PET imaging parameters, semi-automated quantitative analyses were performed using volume-of-interests (VOI). A spherical VOI was drawn to include the primary tumor lesion. Metabolically active tumor was segmented by an adaptive threshold according to tumor and background intensities 19. To consider different machine and reconstruction parameters, standardized uptake value (SUV) of the tumor was corrected by normal liver uptake. A manually drawn spherical volume-of-interest on the right liver was used as a reference liver SUV. The maximum SUV values of the tumor were divided by mean SUV of liver (tumor-to-liver ratio, TLRmax). Metabolic tumor volume of each lesion was calculated by the tumor segmentation on FDG PET. PET data with metabolic tumor volume less than 5 cm^3^ were excluded for further analyses due to underestimation of TLRmax for small sized tumors. The semi-automated quantitative analysis was performed on LifeX software (https://www.lifexsoft.org/, version 4.0) 20.

### scRNA-seq data preparation and clustering

We obtained a read count matrix for the droplet-based scRNA-seq data of 5 lung cancer patients 21. The authors provided filtered cells by removing cells all that had either fewer than 201 unique molecular identifiers, over 6,000 or below 101 expressed genes, or over 10% unique molecular identifiers derived from the mitochondrial genome. The read counts of 52,698 cells were downloaded from the authors' online resource (https://gbiomed.kuleuven.be/scRNAseq-NSCLC). The data were scaled by log-normalization after the read counts divided by total number of transcripts and multiplied by 10,000. Highly variable 2150 genes were selected using *FindVariableGenes* function of Seurat (version 2.3.4) 22. Data were then scaled to z-scores with regressing out of total cellular read counts and mitochondrial read counts. Cell types were determined by the graph-based clustering approach implemented in *FindClusters* function. Before the clustering, dimension reduction was performed by principal component analysis and 50 dimensions were used for the clustering. The conservative resolution was set to 0.3 and the parameter eps was set to 0.5. To identify the marker genes of the clusters, *FindMarkers* function of Seurat package was used and 10 high-ranked marker genes according to the fold-change were identified. The scRNA-seq data were embedded by two-dimensional projection, t-distributed stochastic neighborhood embedding (t-SNE). The method for glucose metabolism profiles of single cell data is described in Supplementary Methods.

### Immunotherapy response prediction using glucose transporter profiles

RNA-seq data of melanoma specimens from patients treated with anti-PD1 therapy (pembrolizumb or nivolumab) were downloaded from the Gene Expression Omnibus (accession number GSE91061) 23. The data included gene expression profiles of 51 baseline tumors and 58 on-treatment tumors. Biopsy samples were obtained before the anti-PD1 therapy (1-7 days before the first dose) and repeated on cycle 1, day 29 (between days 23-29) after starting the treatment. The read counts were scaled by log normalization and GLUT-ratio, the ratio of GLUT3 to GLUT1, was calculated for all samples. ImmuneScore of the melanoma samples was also estimated by xCell as LUSC samples. The association between ImmuneScore and GLUT-ratio was analyzed and we tested whether GLUT-ratio was different between anti-PD1 responders and nonresponders. Patients were divided into two groups according to the response criteria, responders and nonresponders, defined by the irRECIST criteria 24. Nonresponders included patients with progressive disease (PD) and stable disease (SD) after the anti-PD1 therapy. Responders included patients who showed complete or partial response (CR/PR) after the treatment. Another RNA-seq data of melanoma tissues for anti-CTLA4 therapy were additionally downloaded from the Gene Expression Omnibus (accession number GSE115821) 25. 42 baseline gene expression data with scaled by z-score were obtained and GLUT-ratio was also obtained. Of note, gene expression values of anti-CTLA4 data were represented by z-scores, GLUT-ratio was differently defined for this cohort compared with other cohorts in this study: z-score of GLUT3 - z-score of GLUT1 expression. The GLUT-ratio of baseline melanoma was associated with ImmuneScore, anti-CTLA4 response and progression-free survival.

### Statistical analysis

All statistical analyses were performed using the R software package, version 3.4.3 (http://www.R-project.org). The correlation between variables was evaluated by the Pearson's correlation analysis. GLUTs and glycolysis enrichment scores of immune-rich and immune-poor clusters were compared using the independent t-test. The comparison of grade of immunohistochemistry was performed by Kruskal-Wallis test. Comparison of the GLUT-ratio of responders and nonresponders was performed by the Mann-Whitney test. When the three response groups were applied (PD, SD, and PR/CR), the comparison of GLUTratio was performed by Kruskal-Wallis test. Survival analyses were performed using Cox proportional hazards regression. We divided the patients into two groups by the mean value of the GLUT-ratio (high and low GLUT-ratio), and performed survival analysis with the Kaplan-Meier method and the log-rank test.

## Results

### Cancer cells and immune cells in TME express different GLUTs

**Figure 1:**
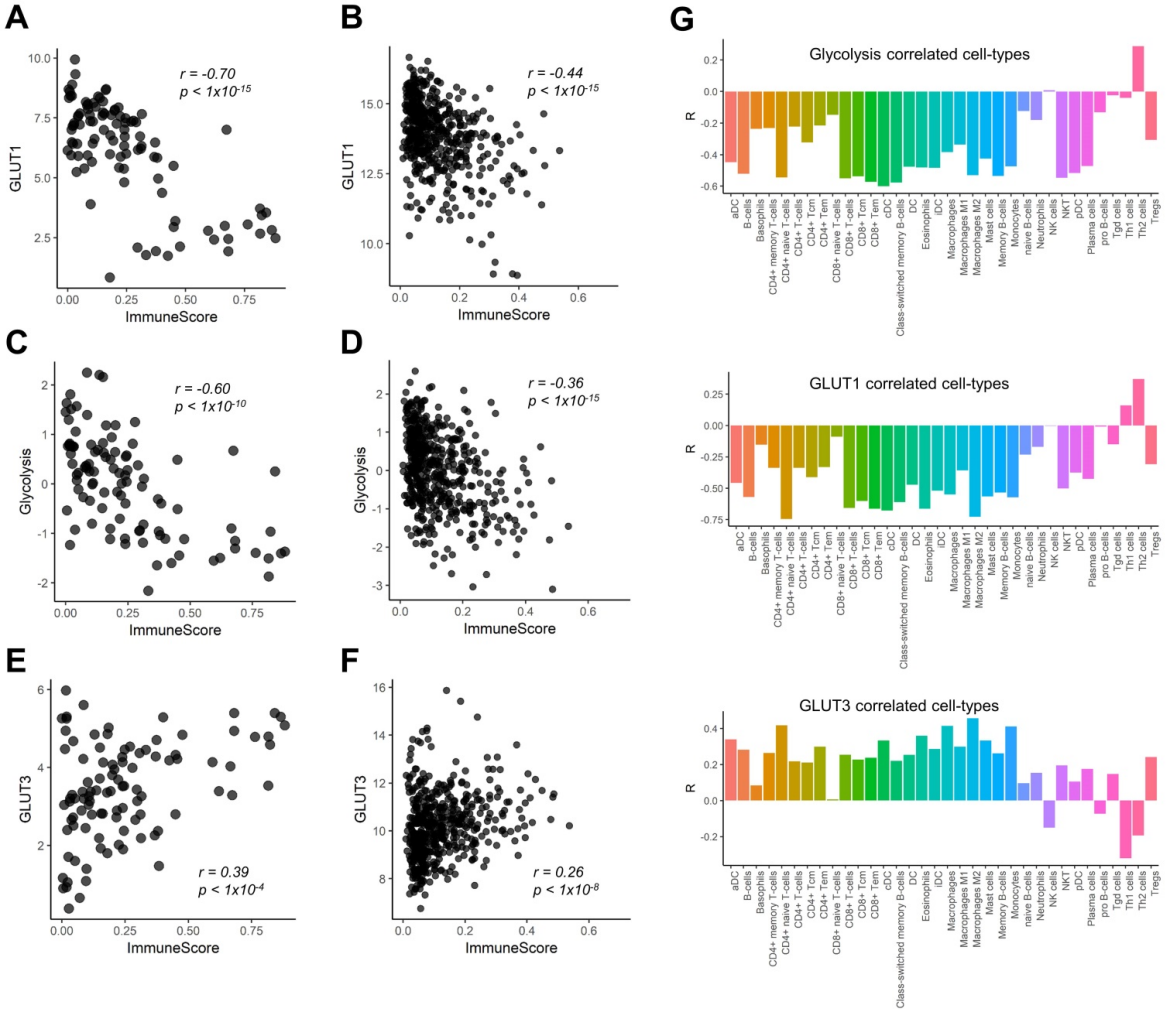
** Correlation of immune cell enrichment score with glucose transporters and glycolysis enrichment score.** The overall immune enrichment score (ImmuneScore) was estimated from the RNA-sequencing data, glycolysis enrichment score was estimated by single sample gene set enrichment analysis from RNA-sequencing data of human lung squamous cell carcinoma. (**A, B**) The expression level of glucose transporter 1 (GLUT1) showed significant negative correlation with ImmuneScore in our study cohort (*r =* -0.70, *p <* 0.001) (A) as well as TCGA cohort (*r =* -0.44, *p <* 0.001) (B). (**C, D**) The association between glycolysis enrichment score and ImmuneScore was similar to that of GLUT1 (*r =* -0.60, *p <* 0.001 in our study cohort; *r =* -0.36, *p <* 0.001 in TCGA cohort). (**E, F**) On the contrary, the expression level of GLUT3 showed significant positive correlation (*r =* 0.39, *p <* 0.001) in our study cohort (E) and in TCGA cohort (*r =* 0.26, *p <* 0.001) (F). (**G**) The enrichment score of each immune cell subtype of tumor microenvironment (TME) showed that most immune cell subtypes showed a significant negative correlation with GLUT1 expression level and glycolysis enrichment score. However, the enrichment scores of most cell subtypes in TME showed a significant positive correlation with GLUT3 expression.

We found a negative correlation between GLUT1 expression and ImmuneScore of LUSC (*r =* -0.70, *p <* 0.001; Figure 1A). This finding was reproduced in the Cancer Genome Atlas (TCGA) data of LUSC (*r =* -0.44, *p <* 0.001; Figure 1B). Considering the metabolic competition based on aerobic glycolysis of immune cells and cancer cells in TME 2, 3, 6, we also examined the relationship between the ImmuneScore and glycolysis enrichment score. They also showed significant negative correlations (*r =* -0.60, *p <* 0.001 in our study cohort; *r =* -0.36, *p <* 0.001 in TCGA cohort Figure 1C, D). In contrast, GLUT3 showed a positive correlation with the ImmuneScore in both cohorts (*r =* 0.39, *p <* 0.001 and *r =* 0.26, *p <* 0.001; Figure 1E, F). We further investigated the specific types of immune cells being associated with GLUTs and glycolysis enrichment score. The Pearson's correlation coefficients were calculated for the enrichment score of each immune cell subtype and glucose metabolic profiles (Figure 1G). Most immune cell subtypes including macrophages and CD8+ T-cells were negatively correlated with GLUT1 and glycolysis enrichment score, while positively correlated with GLUT3.

**Figure 2:**
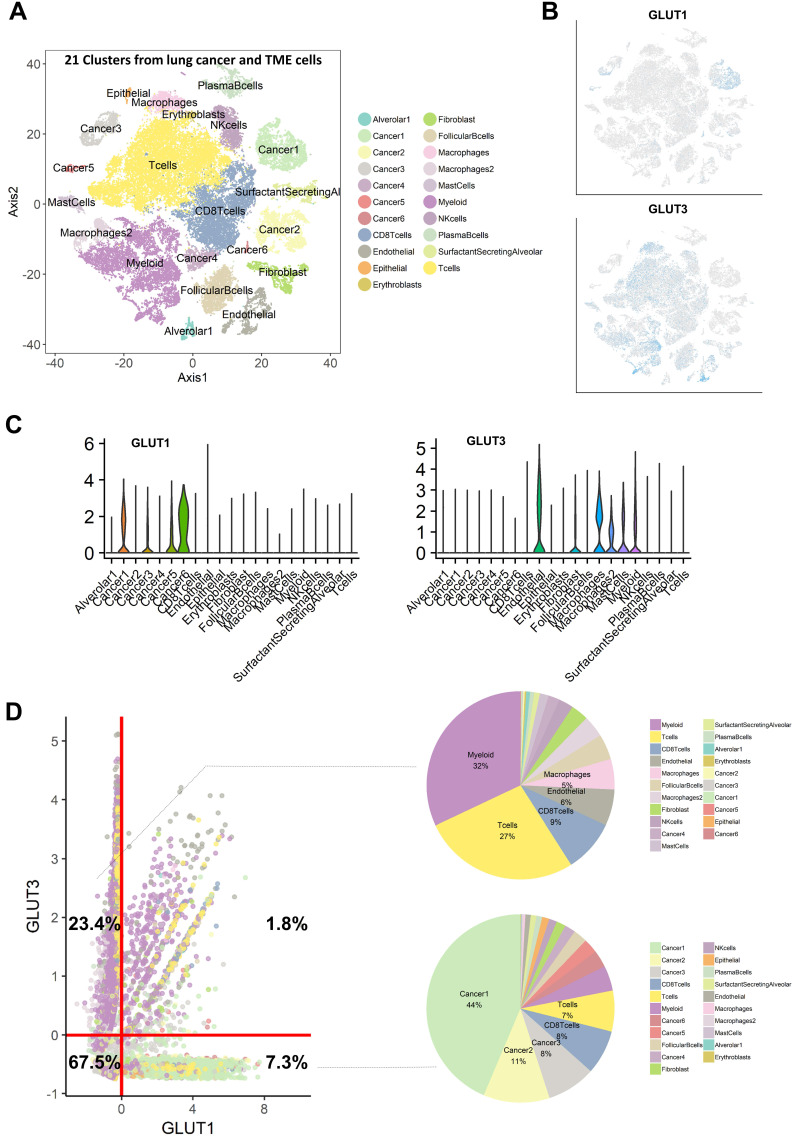
** Single cell RNA-sequencing analysis reveals that GLUT1 and GLUT3 are mainly expressed in cancer cells and immune cells, respectively.** (**A**) t-distributed stochastic neighbor embedding (t-SNE) of the 52,698 single cells are demonstrated with each cell color-coded for the associated 21 cell type clusters. (**B**) The expression of GLUT1 and GLUT3 were depicted on t-SNE plot. GLUT1 is mostly expressed in cancer cell-related clusters, while GLUT3 is mostly expressed in myeloid and T-cell clusters. (**C**) The expression level of GLUT1 and GLUT3 according to the cell type clusters are demonstrated in boxplots. GLUT1 showed high expression level in cancer-related clusters, while other clusters showed negligible expression of GLUT1. Conversely, GLUT3 showed high expression in mostly myeloid cell, epithelial cell, fibroblast cell clusters, while cancer-related clusters showed negligible expression of GLUT3. (**D**) Scatterplot of all 52,698 cells according to the normalized expression level of GLUT1 (x-axis) and GLUT3 (y-axis). Each cell type cluster is overlaid in different colors. The red line depicts the cut off value of normalized single-cell RNA sequencing data. Cells that highly express GLUT3 (23.4%) mainly include myeloid and T-cells express GLUT3, while cells with highly express GLUT1 (7.3%) mainly include cancer cells. There are a very small proportion of cells (1.8%) which expressed both GLUT1 and GLUT3.

We then hypothesized that cancer cells and immune cells in TME would be dependent on different GLUTs, GLUT1 and GLUT3, respectively. Immunohistochemistry of LUSC showed that GLUT3 expression was mainly found on CD3 positive cells, while GLUT1 showed different expression patterns. GLUT3 expression was not found on cancer cell portions in all samples (Figure S1A, B). Additionally, LUSC with low CD3 expression showed no GLUT3 expression (Figure S1C). CD3 expression was found on tumor margin only or stroma in central tumors and tumor margin. The spatial expression pattern of GLUT3 corresponded to CD3 expression patterns (Figure S1D). To corroborate this association, scRNA-seq data of non-small cell lung cancer were utilized 21. Using cell-type specific marker genes, names of clusters were determined (Figure S2). t-SNE plots color-coded for GLUTs highlighted different expression patterns of GLUT1 and GLUT3 (Figure 2A, B). GLUT1 was mostly expressed in cancer cell clusters, while GLUT3 was mainly expressed in myeloid cells, T-cells, and endothelial cells (Figure 2C). All cells were plotted by the two features, GLUT1 and GLUT3 expression level, which explained that cells selectively expressed either GLUT1 or GLUT3, and only few cells expressed both GLUTs (Figure 2D). The GLUT1^+^GLUT3^-^ cells were 7.3% of all cells and mainly included the cancer cell clusters. The GLUT1^-^GLUT3^+^ cells were 23.4% of all cells and more than half of these cells were myeloid and T-cells (Figure 2D). The glycolysis enrichment score of each cell was calculated and cancer cells showed relatively higher glycolysis activity than other cells in TME (Figure S3). The expression levels of GLUTs were compared for subsets of the cells, cancer and immune cells. GLUT1 and glycolysis enrichment score were significantly higher in cancer cells and GLUT3 was significantly higher in immune cells (Figure S3). In addition, most cells in TME expressed very low levels of other GLUTs compared with GLUT1 and GLUT3 (Figure S4). A myeloid subset of TME, the most frequent GLUT1^-^GLUT3^+^ cells, showed a heterogeneous GLUT3 expression. The myeloid cells with high GLUT3 was associated with high glycolysis and low oxidative phosphorylation (Figure S5).

### Glucose uptake of LUSC is influenced by both GLUT1 of cancer cells and GLUT3 of immune cells

**Figure 3:**
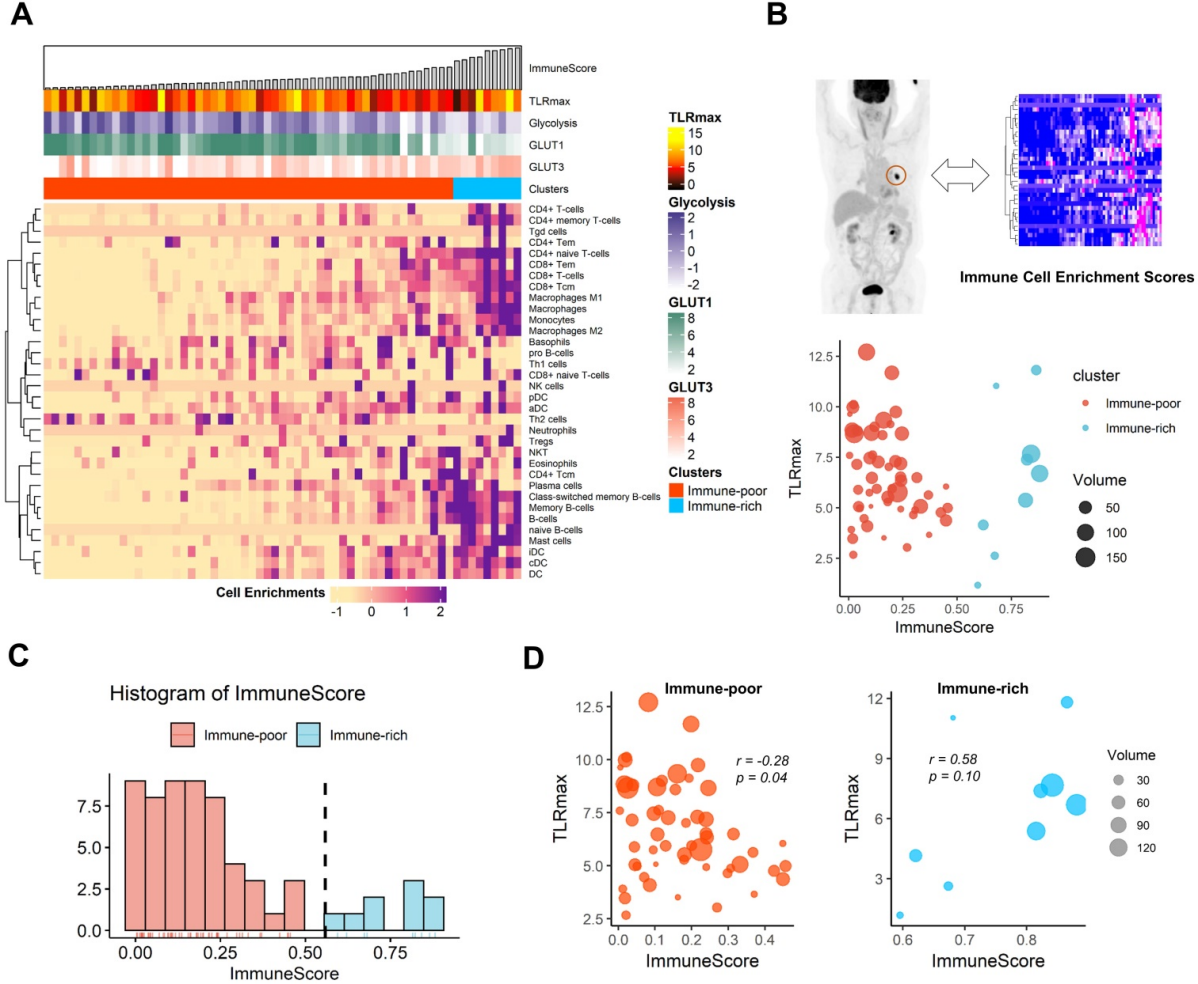
** The association of functional glucose uptake with immune cell enrichment in human lung squamous cell carcinoma.** (**A**) A heatmap depicting immune cell enrichment score of 63 lung squamous cell carcinoma patients. The samples were aligned by ImmuneScore (left to right). The maximum tumor uptake-to-liver ratio (TLRmax) which was measured by ^18^F-Fluorodeoxyglucose positron emission tomography (FDG PET), glycolysis score, GLUT1 and GLUT3 expression level and clusters based on ImmuneScore are shown as for each sample (above the heatmap). (**B**) Scatterplot showing the association of ImmuneScore and TLRmax. The color indicates two distinct clusters based on ImmuneScore, and the size of each spot indicates the tumor volume (cm^3^). (**C**) The distribution of ImmuneScore in the study samples. The samples were divided into two distinct group, immune-rich and immune-poor group. The threshold for the clustering based on the histogram was determined by a kernel density estimation. The threshold was 0.558. (**D**) The correlation between ImmuneScore and TLRmax were depicted separately according to the immune-rich and -poor group. TLRmax showed a negative correlation with ImmuneScore (*r =* -0.28, *p =* 0.04) in immune-poor group. On the contrary, TLRmax showed trend of positive correlation with ImmuneScore in immune-rich group (*r =* 0.58, *p =* 0.10).

The cell-type enrichment scores estimated from tissue RNA-seq data of LUSC were plotted with TLRmax measured on FDG PET as well as expression profiles related to glucose metabolism (Figure 3A). The association between TLRmax and ImmuneScore was complicated according to immune-related clusters of LUSC (Figure 3B). LUSC samples were divided into the two clusters, immune-rich and immune-poor types, according to the histogram of ImmuneScore (Figure 3C). TLRmax was differently correlated with ImmuneScore for these two clusters. In the immune-poor cluster, TLRmax was negatively correlated with ImmuneScore (*r =* -0.28, *p =* 0.04). On the contrary, TLRmax was prone to be positively correlated with ImmuneScore in the immune-rich cluster (*r =* 0.58, *p =* 0.10; Figure 3D). As another dataset, the TLRmax measured from the TCIA data showed a trend to the negative correlation with ImmuneScore (*r =* -0.23, *p =* 0.31). Considering that ImmuneScore of the TCGA samples which had matched FDG PET data in TCIA was relatively lower than the overall range, these samples were included in the immune-poor cluster (Figure S6).

**Figure 4:**
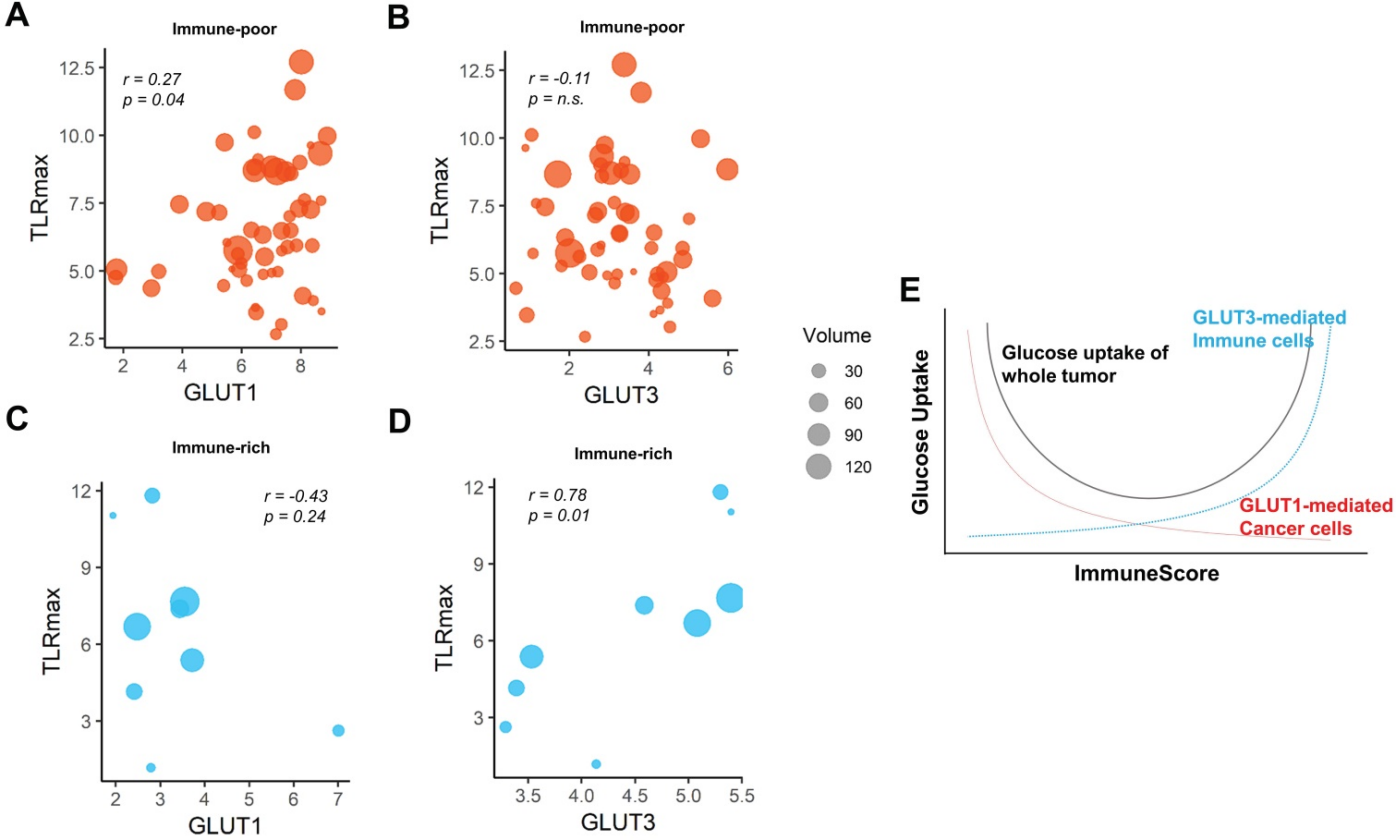
** The pattern of association between TLRmax and glucose transporters were different according to the subgroups based on ImmuneScore.** (**A**) In the immune-poor group, GLUT1 expression showed a positive correlation with TLRmax (*r =* 0.27, *p =* 0.04). (**B**) However, TLRmax showed no correlation with GLUT3 expression in the immune-poor group (*r =* -0.11, *p =* n.s.). (**C**) In immune-rich group, GLUT1 showed a trend of negative correlation with TLRmax, though it did not reach a statistical significance (*r =* -0.43, *p =* 0.24). (**D**) GLUT3 showed a significant positive correlation with TLRmax (*r =* 0.78, *p =* 0.01). (**E**) We propose the association of glucose uptake with immune profiles in TME based on the reciprocal change of glucose metabolism between cancer and immune cells. A schematic diagram shows the overall glucose uptake of tumors mediated by different GLUTs of cancer and immune cells within TME in accordance with the ImmuneScore.

We then investigated whether the different GLUTs would be associated the FDG uptake according to the clusters. For the immune-poor cluster, GLUT1 was positively correlated with TLRmax, while GLUT3 was not (*r =* 0.27, *p =* 0.04 and *r =* -0.11, *p =* n.s., respectively; Figure 4A, B). On the contrary, GLUT1 was prone to be negatively correlated with TLRmax for the immune-rich cluster (*r =* -0.43, *p =* 0.24; Figure 4C). GLUT3 was strongly positively correlated with TLRmax for the immune-rich cluster (*r =* 0.78, *p =* 0.01; Figure 4D). Tumors of immune-poor cluster showed significantly higher GLUT1 than those of immune-rich cluster, while GLUT3 expression was significantly higher in immune-rich cluster than immune-poor cluster. The glycolysis activity was significantly higher for the immune-poor cluster. However, TLRmax was not significantly different for these two clusters (Figure S7). According to the results, we presented the suggested overall association between tumor metabolism and immune cell enrichment in Figure 4E, which showed the glucose uptake of the tumor could be affected by a sum of GLUT1 of cancer cells and GLUT3 of the immune cells within TME.

### The ratio of GLUT3 and GLUT1, a surrogate of the reciprocal glucose metabolic activity between cancer and immune cells, predicts immunotherapy response

**Figure 5:**
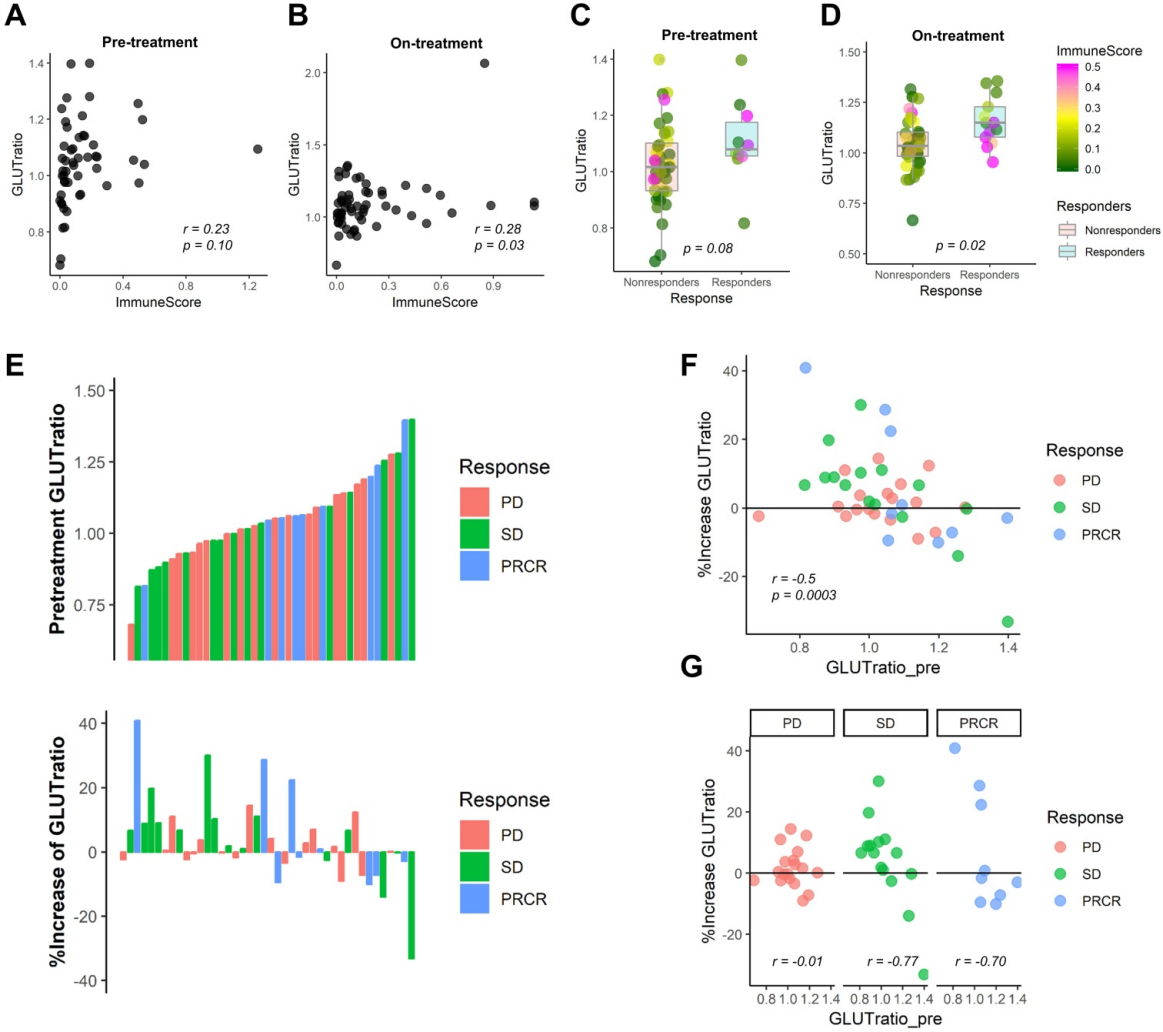
** The ratio of GLUT3 to GLUT1 as a surrogate marker for evaluating metabolic functionality of immune cells in melanoma patients treated with immunotherapy.** From the RNA-sequencing data from melanoma patients who underwent anti-PD-1 treatment, we examined the GLUT-ratio, the ratio of GLUT3 to GLUT1, whether it could predict treatment response. (**A, B**) The GLUT-ratio showed a positive correlation with ImmuneScore in pre-treatment (*r =* 0.23, *p =* 0.10) as well as on-treatment (*r =* 0.28, *p =* 0.03) melanoma tissues. (**C, D**) The GLUT-ratio of nonresponders (stable disease and progressive disease) and responders (complete or partial remission) was compared. (C) There was a trend of higher GLUT-ratio in responders compared with nonresponders in pre-treatment phase (*p =* 0.08). (D) This trend was more prominent in the on-treatment phase. The GLUT-ratio of responders and nonresponders was significantly different in on-treatment phase (*p =* 0.02). (**E**) The relationship between anti-PD-1 treatment response according to RECIST criteria and pre-treatment GLUT-ratio, %change of GLUT-ratio after the immunotherapy treatment was presented. (**F**) A scatter plot depicts a relationship between GLUT-ratio from pre-treatment tissue and the % change of GLUT-ratio after the anti-PD-1 treatment. Overall, there was a significant negative correlation between %change and baseline value of the GLUT-ratio (*r =* -0.50, *p =* 0.0003), which suggests GLUT-ratio is increased after the treatment in tumors with low pretreatment GLUT-ratio. (**G**) According to each response criteria, the patients with SD or PR/CR showed a negative correlation, however, the patients with PD did not show any correlation between two parameters, pretreatment and %change of GLUT-ratio (*r =* -0.77 for SD; *r =* -0.70 for PR/CR; *r =* -0.01 for PD). Specifically, there was very few patients who showed GLUT-ratio increment among low GLUT-ratio in patients with PD. (irRECIST = immune-related response evaluation criteria in solid tumors; C*R =* complete remission; P*R =* partial remission; SD = stable disease; PD = progressive disease).

We expect that the relative metabolic activity of immune cells to cancer cells can be measured by a simple surrogate marker, the ratio of GLUT3 to GLUT1, GLUT-ratio. For melanoma patients who underwent anti-PD1 therapy 23, the GLUT-ratio was positively correlated with ImmuneScore for both pre-treatment and on-treatment data (*r =* 0.23, *p =* 0.10 and *r =* 0.28, *p =* 0.03; Figure 5A, B). The GLUT-ratio of responders tended to be higher than nonresponders before the treatment (*p =* 0.08; Figure 5C). The GLUT-ratio of responders was significantly higher than those of nonresponders in the on-treatment data (*p =* 0.02; Figure 5D). When the patients were divided into three groups according to the response, PR/CR, SD, and PD, the GLUTratio showed a trend of difference according to the response group (Figure S8). Notably, the difference of GLUTratio measured by on-treatment data of the three groups showed a borderline significance (*p =* 0.057, Kruskal-Wallis rank-sum test). A waterfall plot visualizes the association between the response and pre-treatment GLUT-ratio as well as the change of GLUT-ratio during anti-PD1 treatment (Figure 5E). The increase of GLUT-ratio was negatively correlated with pretreatment GLUT-ratio, which suggested that the rise of GLUT-ratio after anti-PD1 was more prominent in tumors with low pre-treatment GLUT-ratio (*r =* -0.50, *p =* 0.0003; Figure 5F). Furthermore, the rise of GLUT-ratio after anti-PD1 in low GLUT ratio tumors was found in patients with SD and CR/PR, while it was not in patients with PD (*r =* -0.77 for SD; *r =* -0.70 for PR/CR; *r =* -0.01 for PD; Figure 5G).

To corroborate the predictive value of GLUT-ratio for immunotherapy response, we investigated the GLUT-ratio in another melanoma data treated with anti-CTLA4 25. The GLUT-ratio was also significantly positively correlated with ImmuneScore (*r =* 0.37, *p =* 0.02; Figure S9A). A trend of higher GLUT-ratio calculated by baseline RNA-seq in tumors of CR/PR than those of PD or SD (*p =* 0.14; Figure S9B). Furthermore, the GLUT-ratio was associated with progression-free survival. The higher GLUT-ratio was significantly associated with a favorable outcome, which suggests clinically good response to anti-CTLA4 (Cox model, hazard ratio = 0.73, 95% confidence interval 0.55-0.98, *p =* 0.04; Figure S9C, D).

## Discussion

In spite of the importance of the metabolic interaction in tumor immunity as well as progression, metabolic characteristics of cancer and immune cells have received poor attention as a clinically feasible biomarker to understand tumor immune functionality. We found that glucose uptake of cancer cells and immune cells in TME were mainly associated with different GLUTs, GLUT1 and GLUT3, respectively. These findings were confirmed by the results of scRNA-seq as well as the correlation of GLUTs with ImmuneScore in LUSC tumors. The function of glucose uptake could be assessed by noninvasive imaging, FDG PET, which showed that uptake was correlated with different GLUTs according to the immune-related subtypes in this study. Though other proteins related to glycolysis, including hexokinase II, also affect FDG uptake, the finding that GLUTs were differently enriched in TME is new considering GLUTs are the main molecule associated with FDG uptake. It suggests that, in viable human tumors, glucose uptake by GLUT1 in cancer cells is relatively suppressed when GLUT3 mediated glucose uptake by immune cells is increased in immune-rich tumors. This competitive glucose metabolism was supported by previous studies in head and neck cancers 26, 27, which showed decreased glucose and FDG uptake according to CD8 T-cell infiltration in TME. Furthermore, among myeloid cells, high GLUT3 myeloid cells were associated with high glycolysis and low oxidative phosphorylation, which suggested anti-tumor population of macrophages 28. It suggested high GLUT3 in immune cells in TME could reflect the enriched anti-tumor immune population. Considering the suppressed aerobic glycolysis in cancer cells by activated effector immune cells in TME, biomarkers which could represent respective glucose metabolism in cancer and immune cells are expected to reflect anti-tumor immunity status.

We assessed the usefulness of the GLUT-ratio to evaluate differential glycolysis activation in cancer and cancer-infiltrating immune cells. The idea was started from our findings that tumors with highly expressed GLUT3 have enriched immune cell infiltration in TME, reflecting metabolically active effector immune cells. Immune cells also use GLUT1 as a glucose transporter, according to previous studies 29, 30. However, our findings suggest that GLUT3 could be a main transporter of immune cells in TME of LUSC, at least. Since the enriched CD8+ T-cells in TME and cytolytic activity are associated with good response to ICI by estimating cellular anti-tumor activity 31, the GLUT-ratio can reflect the active status of tumor immunity as immune effector function requires enhanced aerobic glycolysis 5. Notably, the difference of GLUT-ratio between tumors with responders and non-responders to anti-PD1 was more prominent in the GLUT-ratio measured by on-treatment data. Of note, as shown in the waterfall plot (Figure 5E), some tumors with relatively low pre-treatment GLUT-ratio showed PR/CR or SD, however, GLUT-ratio of these tumors was increased after the treatment. It suggested that GLUT-ratio is possibly used as a dynamic biomarker to evaluate anti-tumor functionality at the early phase of ICI treatment. Activated immune cells which expresses high GLUT3 may be increased after the anti-PD1 treatment, and eventually, promote antitumor immunity. Increased GLUT3 related to increased activation of immune cells during ICI treatment supports a potential mechanism of pseudoprogression, in which increased size of some tumors eventually regressed may reflect increased immune cells within the TME 32. In particular, a few tumors with good response to ICIs showed an increase in FDG uptake at interim response assessment compared with baseline FDG uptake of the tumor 33, 34. These findings have been clinically believed to be caused by increased immune cells, and the GLUT-ratio is expected to clarify the course of tumor after ICIs. In terms of these clinical imaging findings according to ICIs, the GLUT-ratio can be used as a surrogate marker that monitors the changes of functional immunity during ICIs. Even though FDG uptake cannot directly provide immune profiles of TME, it may play a role in tumor characterization if the FDG uptake pattern is interpreted comprehensively with pathology. Furthermore, the differently enriched GLUTs in cancer and immune cells could be a new imaging target for noninvasive assessment of immune profiles 35. A target molecule selectively binding to a GLUT subtype 36 could be used to develop a novel PET imaging method for selective GLUT imaging.

One limitation of our study is that we preliminarily investigated the predictive value of the GLUT-ratio for ICIs in small cohorts limited to melanoma patients. In terms of the anti-tumor activity of tumors, other biomarkers reflect various mechanisms of tumor immune functions such as the immunogenicity, immune resistance as well as effector immune cells 37. Thus, additional methods to integrate biomarkers with different mechanisms are necessary to understand immune status of tumors and develop accurate predictive models for ICIs 37. Regarding RNA-seq data, we used multiple datasets without integration. Different experimental protocols and preprocessing methods could affect the results. Nonetheless, the correlation of GLUTs and ImmuneScore was consistently found. Even though we suggested the close relationship between the GLUT-ratio and immune enrichment in two types of cancers (LUSC and melanoma), we need to validate our hypothesis of the reciprocal glucose metabolism of cancer and immune cells via different GLUTs in other cancer types. A limitation of spatial heterogeneity should be noted. ImmuneScore evaluated by RNA-seq data represented immune enrichment of a small proportion of a tumor, while FDG uptake patterns on PET image represented tumor metabolism at the macro-scale. It implied that heterogeneity of ImmuneScore affects the results of correlation due to spatially different tumor sites of TLRmax and RNA-seq analysis. To clarify the correlation and to solve these issues, spatially co-registered correlative analysis between FDG uptake and transcriptome analyses will be needed. Furthermore, as the association between GLUTs and ImmuneScore was evaluated in LUSC, the predictive value in lung cancer patients should be further validated as future work.

Our integrative approach of multiscale data including bulk and scRNA-seq and clinical functional imaging has the potential to comprehensively understand tumor immune status and dynamic interactions in TME. Our results would be of great interest to clinically understand metabolic features of tumors related to immune functionality, which highlights the role of immune cells of TME in interpreting FDG uptake. Moreover, our findings may be applied to develop another biomarker reflecting metabolic profiles of tumors to predict and monitor patients who would undergo immunotherapy. We expect that our suggested biomarker based on the metabolism of TME will be utilized to understand complicated mechanisms of immune-metabolism interaction associated with immunotherapy.

## Supplementary Material

Supplementary methods, figures, and tables.

## Abbreviations

TME: tumor microenvironment
FDG: fluorodeoxyglucose
LUSC: lung squamous cell carcinoma
GLUT: glucose transporter
ICI: immune checkpoint inhibitor
scRNA-seq: single-cell RNA-sequencing
TCGA: The Cancer Genome Atlas
TCIA: The Cancer Imaging Archive
VOI: volume-of-interests
SUV: standardized uptake value
TLRmax: tumor-to-liver ratio
t-SNE: t-distributed stochastic neighborhood embedding
PD: progressive disease
SD: stable disease
CR/PR: complete or partial response
