# Supplementary material for: Reciprocal change in Glucose metabolism of Cancer and Immune Cells mediated by different Glucose Transporters predicts Immunotherapy response
Source: Theranostics. 2020 Jul 25;10(21):9579–90. doi: 10.7150/thno.48954 (PMC7449929; doi:10.7150/thno.48954)
Supplement: Supplementary file 1 — Supplementary methods, figures, and tables. [file thnov10p9579s1.pdf]

Supplementary Materials

Reciprocal change in glucose metabolism of cancer and immune cells mediated by different glucose transporters predicts immunotherapy response

Supplementary Methods

Supplementary Tables

Supplementary Figures

References for Supplementary Methods

Supplementary Methods

FDG PET imaging

Patients fasted for at least 6 hours before the image acquisition to have blood glucose level less than < 140 mg/dL. FDG (5.18 MBq/kg) was intravenously injected to patients and the emission scan was performed 60 minutes after the injection using dedicated PET/CT scanners (Biograph mCT40 and mCT64, Siemens Healthcare, Germany). The emission scan was obtained from the skull base to the proximal thigh and a CT scan was consecutively obtained for attenuation correction. PET images were reconstructed using an iterative algorithm (ordered-subset expectation maximization) with image matrix size of 256 × 256, iteration number 2 and 21 subsets.

FDG-PET/CT data provided by The Cancer Imaging Archive (TCIA) were also used [1]. Twenty-two patients who had both RNA-seq and FDG PET data were used. FDG-PET/CT images were acquired according to the standard imaging protocol of multiple institutes. PET data were reconstructed by different methods and parameters based on iterative algorithms according to institutes.

Preprocessing of TCGA RNA-sequencing data and concordant FDG PET from TCIA

We used mRNA transcriptome data of LUSC from The Cancer Genome Atlas projects (TCGA) and concordant FDG PET image from TCIA for validation purpose [1]. Using 'TCGABiolinks' R package [2], we downloaded the level three RNA sequence data of lung squamous cell carcinoma from TCGA data portal (https://portal.gdc.cancer.gov/) obtained with Illumina HiSeq RNASeqV2 (Illumina, San Diego, CA, USA). There were 22 TCGA samples which had matched FDG PET data in TCIA. The acquisition and reconstruction parameters such as matrix size were different according to the imaging protocol of institute. FDG PET data of TCIA were analyzed with the same manner described in the previous

section.

Estimation of glycolysis enrichment score

To examine the overall glycolytic activity of tumor microenvironment, we used Reactome to select genes of glycolysis pathway [3]. Single sample gene set enrichment analysis was applied against the curated gene sets of the Reactome glycolysis pathways to define metabolic profiles of each cancer samples. We implemented single sample gene set enrichment analysis[3] using the curated gene sets from canonical pathways (MSigDB C2, Broad Institute; version 3.0) with GSVA R/Bioconductor package [4, 5]. The enrichment scores of Reactome glycolysis pathway was normalized by z-score across all samples.

Glucose metabolism profiles of single cell data

Glucose metabolism profiles of single cell were evaluated by the scaled gene expression data. The gene expression values of glucose transporter families were obtained. All cells were plotted by GLUT1 and GLUT3 expression. The scaled gene expression of GLUTs with z-score > 0 used as a threshold to determine GLUT1- and GLUT3-positive cells. The proportion of four different groups according to GLUT1 positivity and GLUT3 positivity was estimated. The glycolysis enrichment and oxidative phosphorylation of each cell was estimated by *AddModuleScore* function of Seurat package [6]. The gene sets related to glycolysis and oxidative phosphorylation were obtained by Reactome [3].

Immunohistochemistry

For immunohistochemistry of tumor tissues obtained by LUSC patients (n = 9), formalin-fixed, paraffin-embedded surgical tissue specimens were collected and cut with 4 μm thickness. The primary antibodies were used according to the optimized condition: anti-

GLUT1 (#ab652, Abcam; diluted 1:1000), anti-GLUT3 (#ab41525, Abcam; 4 ug/ml), and anti-CD3 (ab5690, Abcam; diluted 1:100). The samples were amplified with avidin-biotin peroxidase complexes and developed using DAB. Then, the samples were counterstained with hematoxylin.

Supplementary Tables

Supplementary Table 1. Demographic and clinical characteristics of 63 lung squamous cell carcinoma patients

Variables	N = 63
Age (years)	67.4 ±8.8
Sex (Male:Female) (n, %)	59 (93.7%) : 4 (6.3%)
Smoking history (n, %)	
Current	21 (33.3%)
Ex	34 (54.0%)
Never	8 (12.7%)
Pathologic stage* (n, %)	
T1/T2/T3/T4	12 / 27 / 17 / 7
N0/N1/N2	34 / 14 / 15

*Based on AJCC 8th edition

Supplementary Figures

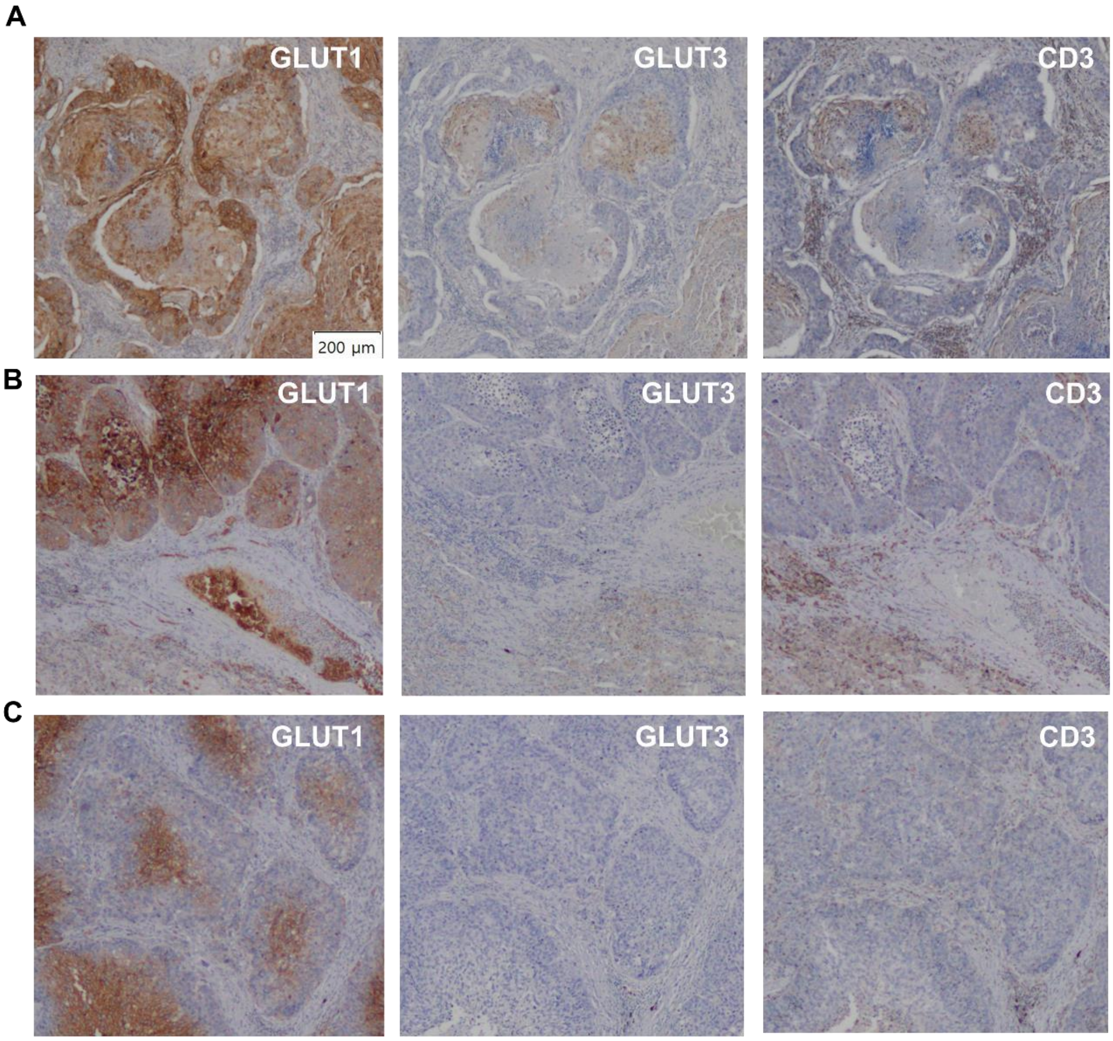

D

	GLUT3 (-)	GLUT3 (+) margin	GLUT3 (+) tumor and margin
CD3 (-)	**1**	**1**	**0**
CD3 (+) margin	**0**	**3**	**0**
CD3 (+) tumor and margin	**0**	**0**	**4**

Supplementary Figure 1. Immunohistochemistry of GLUT1, GLUT3, and CD3 for LUSC. (A) Immunohistochemistry showed GLUT3 expression was found in the proportion of CD3 positive cells in LUSC tissues. (B) Tumor which represented CD3 positive cells mostly in the margin of tumor showed GLUT3 positive cells in the margin. Note that GLUT3 expression on cancer cells was not found. (C) LUSC tissues with low CD3 expression showed low level expression of GLUT3 on immunohistochemistry. (D) Tumors were divided into three groups according to CD3 expression patterns: CD3 negative, CD3 positive in margin, and CD3

positive in central tumor and margin. Accordingly, GLUT3 expression pattern corresponded to the pattern of CD3.

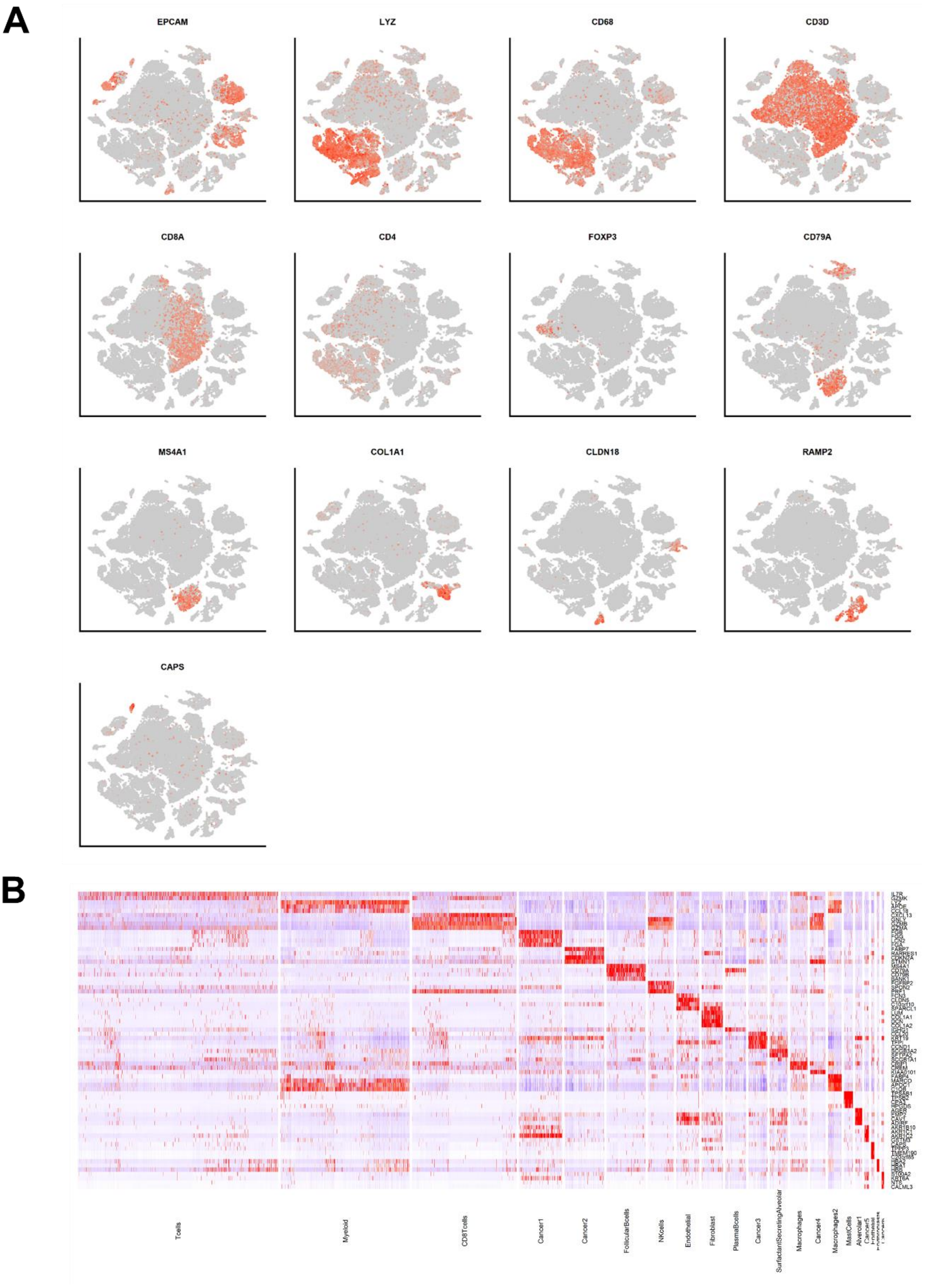

Supplementary Figure 2. **Twenty-one cell type clusters identified from single-cell RNA sequencing data of non-small cell lung cancer.** (A) t-SNE demonstrates clustering of 52,698

cells based on gene expression, and expression of the maker genes for each cluster was overlaid in red color. (B) A heatmap of depicting expression of marker genes according to the 21 cell type clusters. The name of the cell type cluster is annotated at the bottom, and the name of the marker genes are annotated on the right.

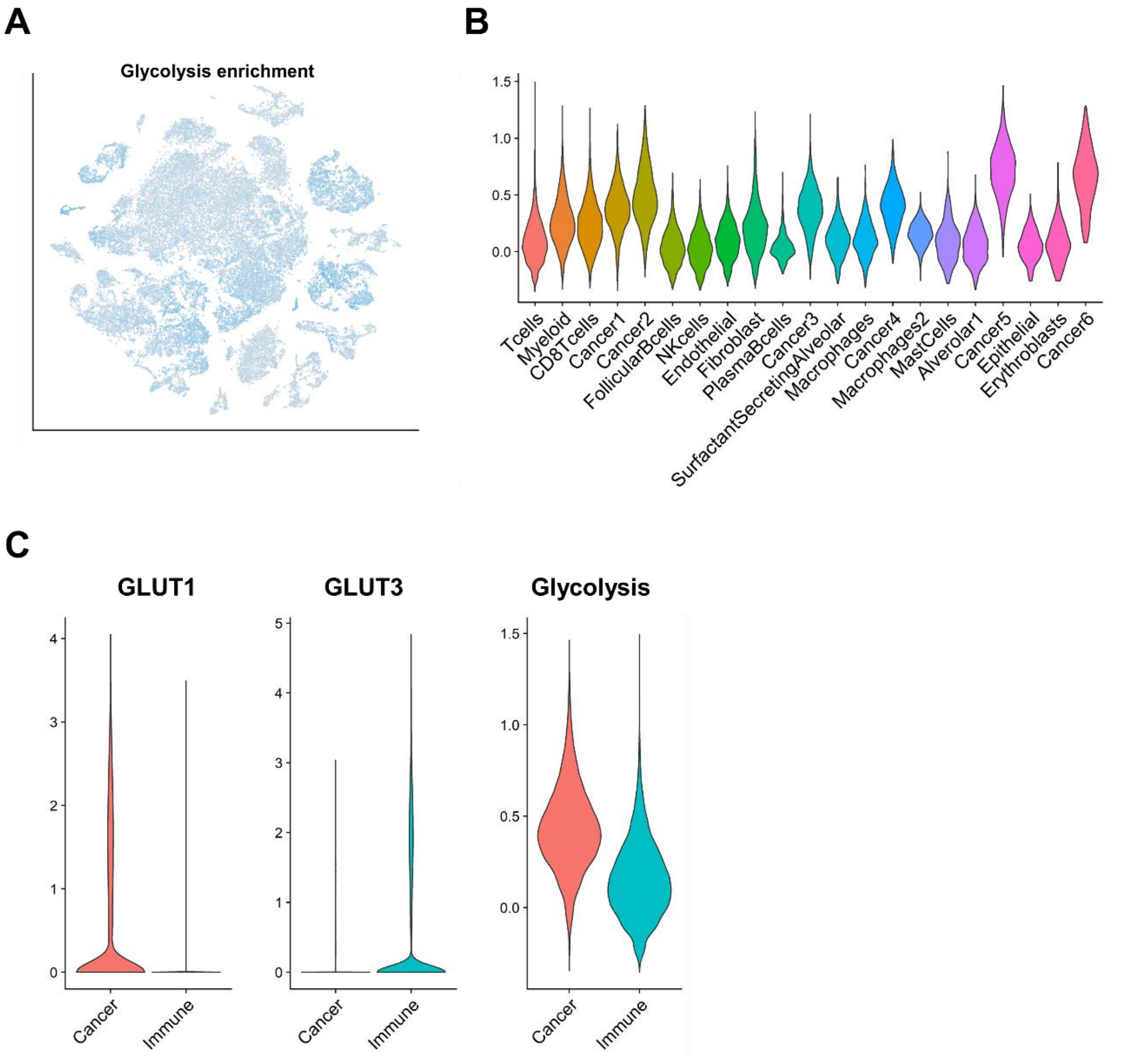

Supplementary Figure 3. Glycolysis score of each cell type cluster. The glycolysis score was estimated from the single-cell RNA sequencing data using single sample gene set enrichment analysis. (A) Glycolysis enrichment score of each cell was drawn for a color-coded t-SNE plot. (B) Cancer cell-related clusters showed higher glycolysis score than other clusters. (C) Cell types were reclassified as cancer and immune cells. GLUT1 expression was significantly higher in cancer cells, while GLUT3 expression was significantly higher in immune cells ($U = 2.1 \times 10^{8}$, $p < 1 \times 10^{-15}$ for GLUT1, $U = 1.3 \times 10^{8}$, $p < 1 \times 10^{-15}$ for GLUT3). Glycolysis enrichment score was significantly higher in cancer cells ($U = 2.6 \times 10^{8}$, $p < 1 \times 10^{-15}$).

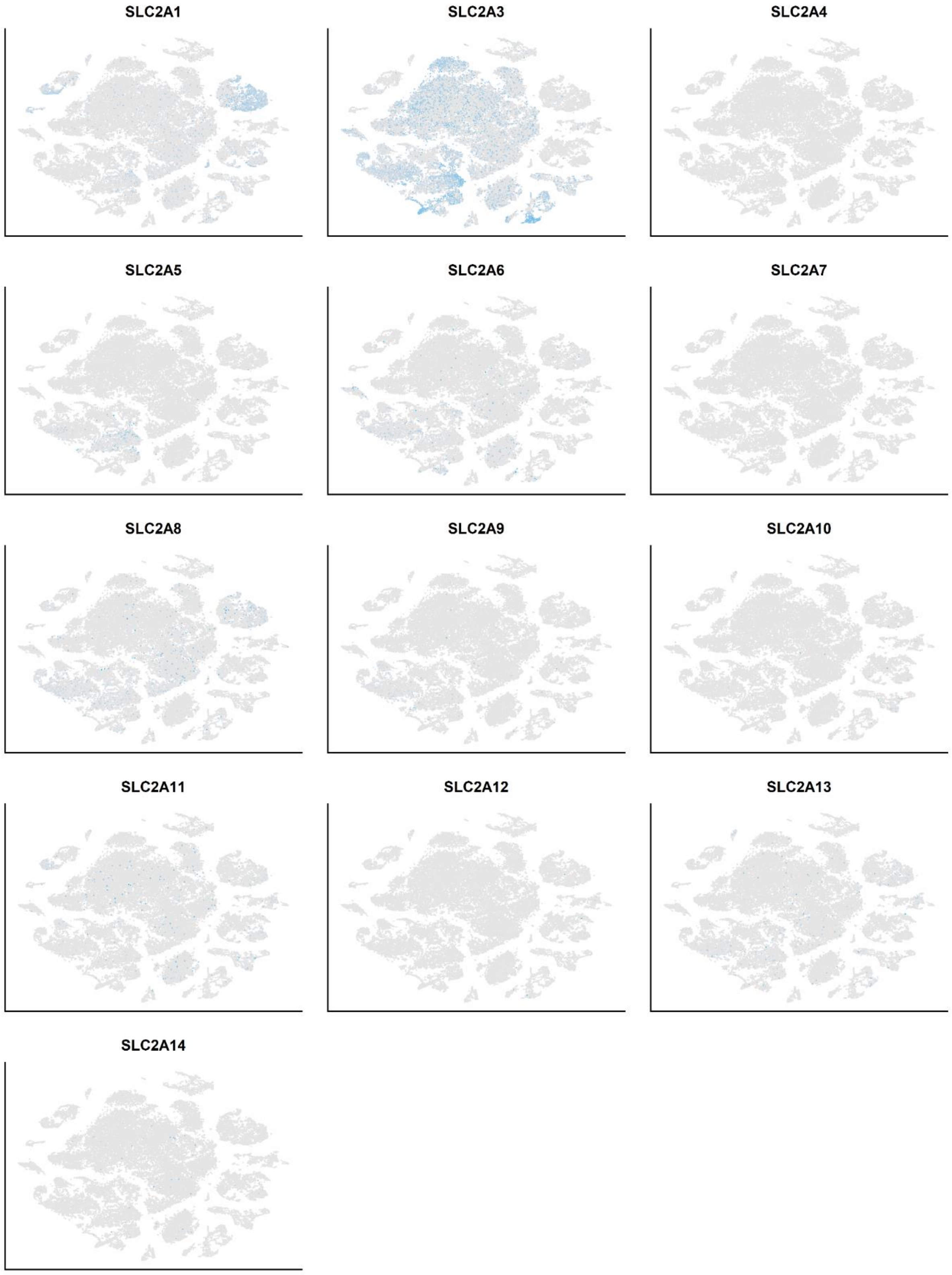

Supplementary Figure 4. Expression of all glucose transporter subtype in cell-type clusters. GLUT1 was mostly expressed in cancer-related clusters and GLUT3 was mostly

expressed in myeloid cell types. Other GLUT subtypes (GLUT4-14) were relatively expressed in low level across many clusters and they did not show patterns of cell specificity. The expression level of GLUT2 was too low in single cells to depict in the t-SNE plot.

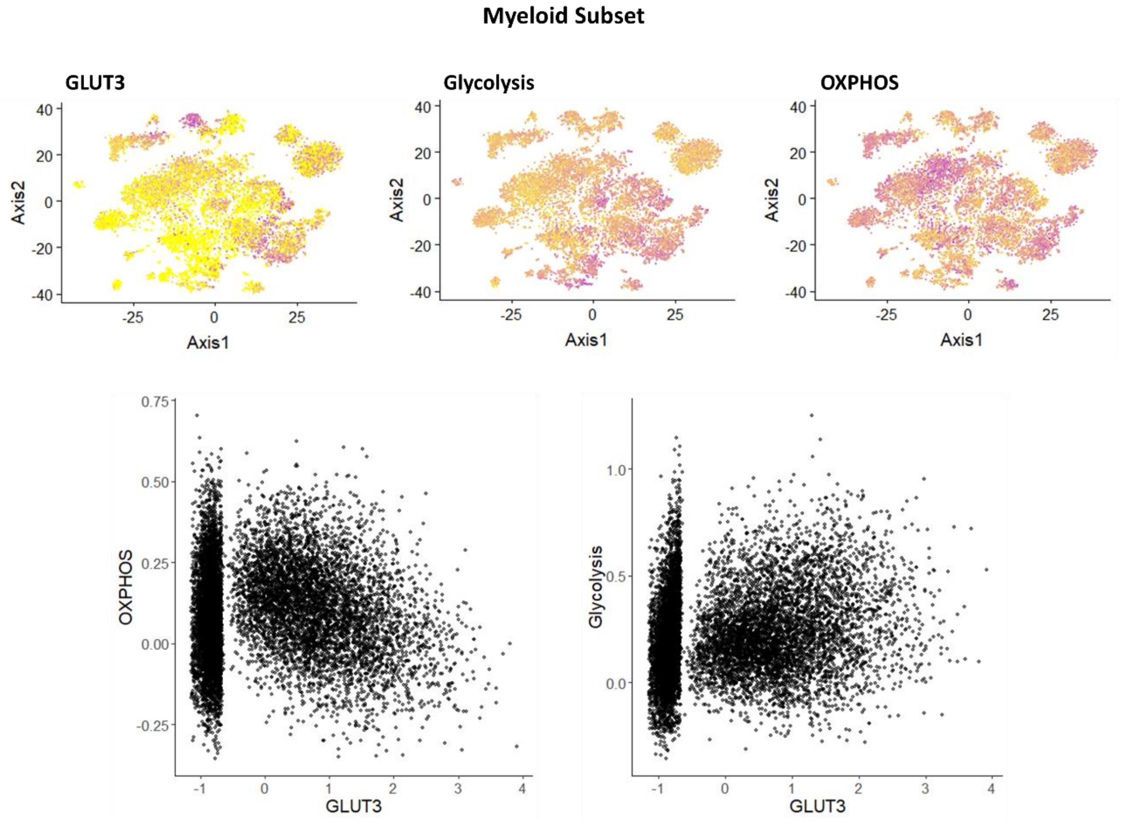

Supplementary Figure 5. A myeloid subset of TME and features related to glucose metabolism. Myeloid cells were the most frequent cells of TME that showed high GLUT3 and low GLUT1. Among myeloid cells, features of glucose metabolism including GLUT3, glycolysis and oxidative phosphorylation (OXPHOS) were evaluated. The myeloid cells with high GLUT3 was associated with high glycolysis and low OXPHOS.

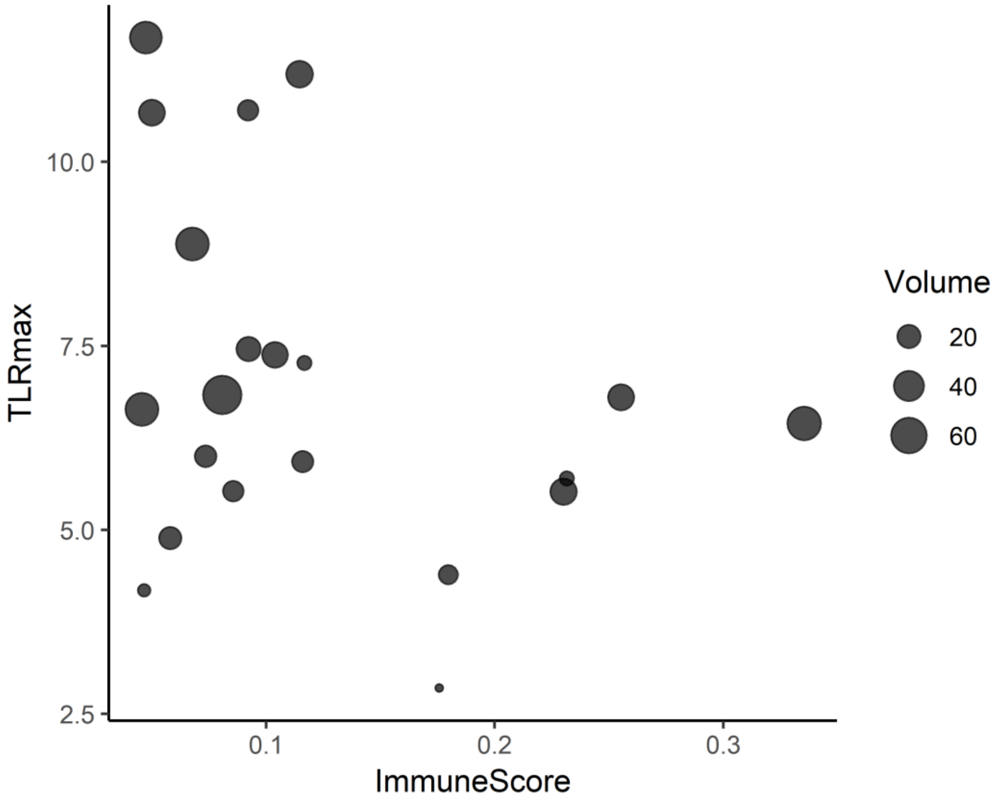

Supplementary Figure 6. Correlation between ImmuneScore and FDG uptake in TCGA cohort. Lung squamous cell carcinoma samples from TCGA cohort included corresponding baseline FDG PET images in TCIA. The TLRmax was measured from PET image and it showed a negative correlation with ImmuneScore without statistical significance (rho = -0.23, p = 0.31). (TLRmax = maximum tumor uptake-to-liver ratio)

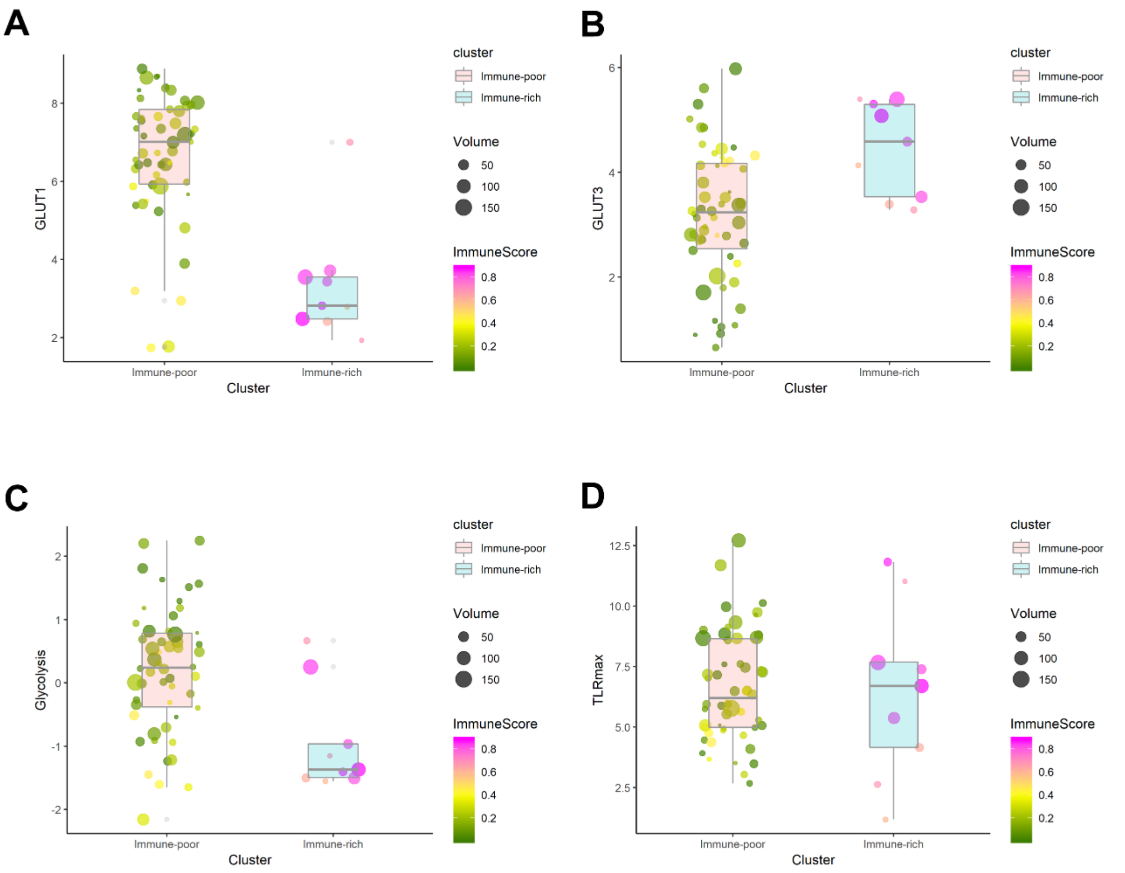

Supplementary Figure 7. Difference in expression of glucose transporters and glycolysis between immune-rich and -poor groups. (A, B) Immune-rich group showed significantly lower expression of GLUT1 (mean GLUT1 expression 6.68 ± 1.64 vs. 3.35 ± 1.49, Immune-poor and Immune-rich, respectively; t = 6.10, p < 0.001), however, it showed significantly higher expression of GLUT3 (mean GLUT3 expression 3.20 ± 1.27 vs 4.46 ± 0.89, Immune-poor and Immune-rich, respectively; t = -3.67, p = 0.003). (C) Glycolysis enrichment score is significantly higher in immune-poor group compared to immune-rich group (glycolysis enrichment score 0.20 ± 0.98 vs -0.95 ± 0.83, Immune-poor and Immune-rich, respectively; t = 3.7377, p = 0.003). (D) TLRmax showed no difference between two groups (TLRmax 6.62 ± 2.25 vs. 6.44 ± 3.55, Immune-poor and Immune-rich, respectively, t = 0.15, p = 0.885).

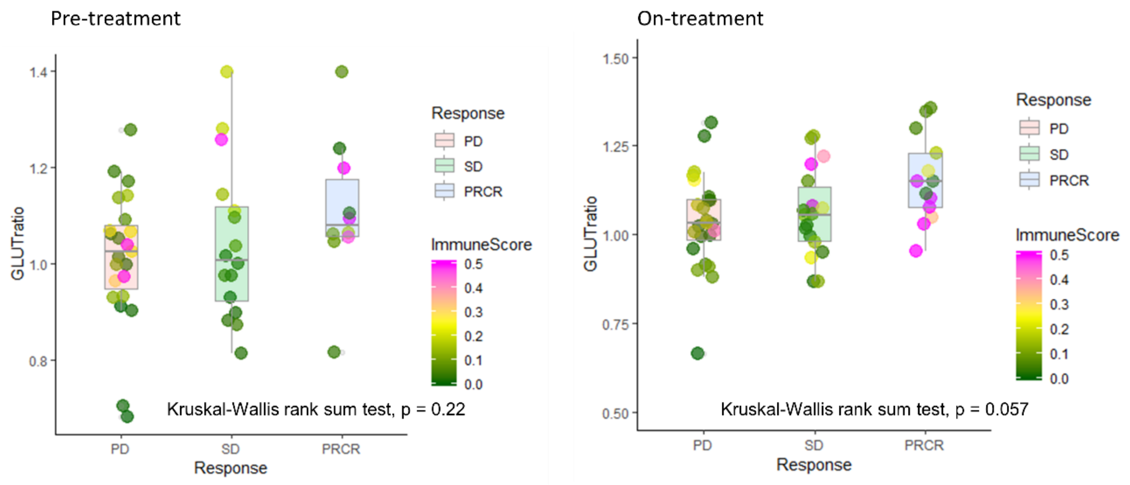

Supplementary Fig. 8. The ratio of GLUT3 to GLUT1 according to the treatment response in the melanoma cohort. Patients were divided into three groups, PD, SD and CR/PR, according to the response, the GLUTratio was compared. Similar to the analysis based on the two groups (responders/nonresponders), a trend of higher GLUTratio in PR/CR group was identified, though the difference of GLUTratio was not statistically significant. Note that the difference was more prominent in the data of on-treatment.

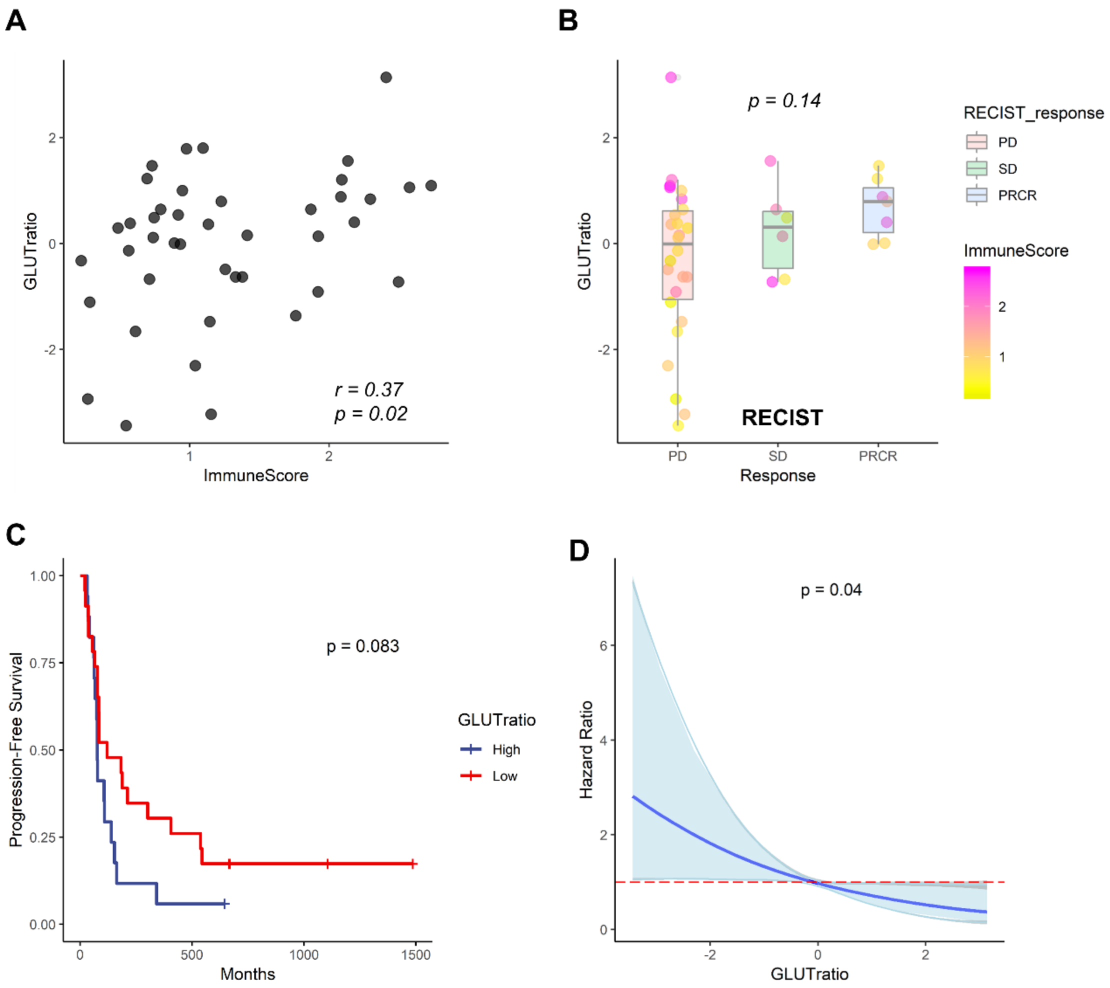

Supplementary Figure 9. Evaluation of the GLUT-ratio as a surrogate marker for anti-CTLA4 therapy as well as prognosis. From the RNA-sequencing data acquired from the 42 melanoma patients who underwent anti-CTLA4 treatment, we evaluated the performance of the GLUT-ratio for immunotherapy response and prognostic factor. (A) The GLUT-ratio showed significant positive correlation in ImmuneScore (r = 0.37, p = 0.02) which was consistent with our previous observations. (B) The patients were divided into three groups according to RECIST criteria and the GLUT-ratio was compared between the groups. There was a trend of increasing GLUT-ratio according to the response (PR/CR group = 0.68 ± 0.57 ; SD group = 0.24 ± 0.87; PD group = -0.32 ± 1.52 ; chi-square = 3.88, p = 0.14). (C) We divided this cohort into two groups according to a cutoff GLUT-ratio using the mean value (high/low

GLUT-ratio group), and the high GLUT-ratio group showed a trend of better progression-free survival ($p = 0.083$) after anti-CTLA4 treatment. (D) As a continuous variable, GLUT-ratio was significantly associated with progression, increased GLUT-ratio was associated with good prognosis (Cox hazard ratio associated with GLUT-ratio, hazard ratio = 0.73, 95% confidence interval 0.55-0.98, $p = 0.04$). (RECIST = response evaluation criteria in solid tumors; CR = complete remission; PR = partial remission; SD = stable disease; PD = progressive disease)

REFERENCES

1. Clark K, Vendt B, Smith K, Freymann J, Kirby J, Koppel P, et al. The Cancer Imaging Archive (TCIA): maintaining and operating a public information repository. J Digit Imaging. 2013;26:1045-57.
2. Colaprico A, Silva TC, Olsen C, Garofano L, Cava C, Garolini D, et al. TCGAbiolinks: an R/Bioconductor package for integrative analysis of TCGA data. *Nucleic Acids Res*. 2016;44:e71.
3. Croft D, Mundo AF, Haw R, Milacic M, Weiser J, Wu G, et al. The Reactome pathway knowledgebase. *Nucleic Acids Res*. 2014;42:D472-7.
4. Liberzon A, Subramanian A, Pinchback R, Thorvaldsdóttir H, Tamayo P, Mesirov JP. Molecular signatures database (MSigDB) 3.0. *Bioinformatics*. 2011;27:1739-40.
5. Hänzelmann S, Castelo R, Guinney J. GSVA: gene set variation analysis for microarray and RNA-seq data. *BMC bioinformatics*. 2013;14:7.
6. Satija R, Farrell JA, Gennert D, Schier AF, Regev A. Spatial reconstruction of single-cell gene expression data. *Nat Biotechnol*. 2015;33:495-502.
